# Problemas Relacionados à Trombocitopenia em Pacientes com Fibrilação Atrial Concomitante que Necessitam de Prevenção Antitrombótica: Um Estudo de Coorte Retrospectivo

**DOI:** 10.36660/abc.20190599

**Published:** 2020-10-13

**Authors:** Renato De Vecchis, Andrea Paccone, Silvia Soreca

**Affiliations:** 1 DSB 29 “S. Gennaro dei Poveri Hospital” Medical and Polyspecialist Centre Naples Itália Medical and Polyspecialist Centre, DSB 29 “S. Gennaro dei Poveri Hospital”, Naples - Itália; 2 University of Bari “Aldo Moro” Department of Cardiology Bari Itália University of Bari “Aldo Moro” - Department of Cardiology, Bari – Itália

**Keywords:** Estudos de Coortes, Trombocitopenia, Fibrilação Atrial, Anticoagulantes, Edoxabana, Enoxaparina, Hemorragia, Acidente Vascular Cerebral, Cirrose Hepática Heparina/uso terapêutico

## Abstract

Baixas doses de edoxabana e enoxaparina sódica foram objeto de uma comparação retrospectiva implementada com a técnica do escore de propensão a fim de mitigar os efeitos das diferenças nas características clínicas basais de duas coortes e minimizar o risco de viés.

Posteriormente, usando um modelo de riscos proporcionais de Cox, avaliou-se a associação de cada tipo de terapia com o risco do composto de morte por todas as causas, acidente vascular cerebral/ataque isquêmico transitório, hospitalizações e ocorrência de sangramentos maiores. Para essa análise, um valor de p < 0,05 foi considerado estatisticamente significante. A terapia com enoxaparina e cirrose hepática como causadora de trombocitopenia estiveram associadas ao aumento do risco do endpoint composto (enoxaparina: hazard ratio (HR): 3,31; IC 95%: 1,54 a 7,13; p = 0,0023; cirrose hepática, HR: 1,04; 95% CI: 1,002 a 1,089; p = 0,0410). Por outro lado, a terapia com edoxabana mostrou-se significativamente associada à diminuição do risco do endpoint composto (HR: 0,071; 95% CI: 0,013 a 0,373; p = 0,0019). Com base nessa análise retrospectiva, o edoxaban em doses baixas seria uma ferramenta farmacológica segura e eficaz para a profilaxia de eventos cardioembólicos em pacientes com FA e trombocitopenia.

## Introdução

Um problema comum é a presença de fibrilação atrial (FA) em pacientes que sofrem de trombocitopenia, o que obviamente contraindica a administração de fármacos anticoagulantes em dose completa.^1^ Além disso, nesses casos, a implementação de um programa de ablação de FA envolve risco não desprezível, visto que os três primeiros meses após a ablação, o chamado “período de supressão”, coincide com a necessidade de administrar, dado o alto risco de recidivas de FA, não apenas antiarrítmicos, mas também anticoagulantes em doses completas.^2^ Em vez disso, a administração de heparina fracionada de baixo peso molecular, ou seja, enoxaparina sódica^3^ na dose de 4000 UI por dia, surgiu como uma opção viável. Alternativamente, uma abordagem terapêutica amplamente praticada foi o antagonista da vitamina K edoxabana^4^ na dose prefixada de 30 mg por dia.

No presente estudo de coorte retrospectiva, que abrangeu um período mediano de 40 meses (intervalo interquartil: de 36 a 48 meses), 220 pacientes foram incluídos de acordo com um critério arbitrário, ou seja, sem realizar cálculos de potência e de tamanho amostral. No total, os pacientes submetidos a edoxabana foram 90, ao passo que 130 pacientes foram submetidos a enoxaparina.

Os requisitos para inclusão no estudo retrospectivo foram: trombocitopenia moderada, definida por uma concentração plaquetária entre 99.000 e 30.000 trombócitos por mm^3^; FA crônica, sujeita à estratégia de controle de taxas; ausência de FA paroxística recém-diagnosticada.

O recrutamento de casos se baseou na constituição de grupos homogêneos, de acordo com o método do escore de propensão de pareamento (*propensity score matching*)^5^ para diminuir o risco de viés resultante das diferenças nas características clínicas basais dos dois grupos. Os pacientes que estavam tomando heparina não fracionada foram pareados com uma dose baixa (30 mg por dia) de edoxabana com características clínicas basais o mais semelhante possível, com uma proporção de 1:1. Aplicamos um modelo de regressão logística. Constatou-se que diversas variáveis estiveram significativamente associadas à probabilidade de pertencer a um dos grupos, com base na metodologia de eliminação *backward stepwise* (ponto de corte p = 0,05). As seguintes variáveis foram usadas para calcular o escore de propensão para cada paciente: idade e nível de cuidado na data índice, uso anterior de anticoagulantes, uso de fármacos antianginosos, uso de insulina, acidente vascular cerebral, custos de hospitalização no período basal. Os pacientes foram pareados na base de 1:1 dentro dos grupos etários de 5 anos específicos para o sexo, com base no escore de propensão com uma diferença máxima permitida de 0,001.

A análise estatística adotada posteriormente foi a construção de um modelo de regressão de risco proporcional de Cox. Escolheu um *endpoint* composto que incluiu morte por todas as causas, acidente vascular cerebral/ataque isquêmico transitório, hospitalizações e sangramentos maiores. As variáveis de exposição levadas em consideração foram: terapia com edoxabana de baixa dosagem, terapia com enoxaparina, hipertensão, diâmetro anteroposterior do átrio esquerdo > 40 mm, fração de ejeção do ventrículo esquerdo < 40%, idade > 85 anos, cirrose hepática como causa de trombocitopenia, doença de Werlhof como causa de trombocitopenia. Em todas as análises estatísticas, o valor p < 0,05 foi considerado estatisticamente significante.

Os cálculos foram feitos usando o Excel 2016 (versão 16.0, Seattle, WA, EUA), além de MedCalc versão 18.6 (Acacialaan 22, 8400 Ostend, Bélgica) e Epi-Info versão 7.1.5.0 para Windows (Centers for Disease Control and Prevention, Atlanta, Geórgia — EUA). O aumento do risco de ser afetado pelo *endpoint* composto mostrou-se associado à exposição à terapia com enoxaparina (*hazard ratio* (HR): 3,31; IC 95%: 1,54 a 7,13; p = 0,0023) e cirrose hepática como causadora de trombocitopenia (HR: 1,04; IC 95%: 1,002 a 1,089; p = 0,0410). Até mesmo a hipertensão esteve associada a risco elevado (HR: 1,104; IC 95%: 1,011 a 1,966; p = 0,0477). Por outro lado, a terapia com edoxabana esteve significativamente associada à diminuição do risco de *endpoint* primário (HR: 0,071; IC 95%: 0,013 a 0,373; p = 0,0019).

A heparina é uma causa não desprezível de trombocitopenia. Esse fato parece se aplicar também à heparina fracionada de baixo peso molecular, ou seja, enoxaparina sódica, a julgar pelos resultados encontrados, que desaconselham o uso da terapia com enoxaparina em pacientes com trombocitopenia documentada. Além disso, a iminente progressão da cirrose hepática pode ter contribuído para causar um risco significativamente maior do *endpoint* composto. De fato, é provável que os sangramentos maiores devidos à ruptura de varizes gastroesofágicas tenham desempenhado um papel substancial na determinação da inferência conclusiva de que o composto é desfavoravelmente influenciado pela cirrose hepática como variável de exposição. Por outro lado, a terapia com edoxabana em doses baixas em pacientes trombocitopênicos mostrou-se protetora contra o composto de morte por todas as causas, acidente vascular cerebral/ataques isquêmicos transitórios, hospitalizações e sangramentos maiores. A principal limitação do estudo é sua natureza retrospectiva, que não permite tirar conclusões definitivas sobre a comparação entre enoxaparina e edoxabana, devido à possibilidade de confusão por indicação, apesar do fato de a técnica de escore de propensão de pareamento ter sido adotada como contramedida. No entanto, com base em nossa análise retrospectiva, o edoxabana em doses baixas seria uma ferramenta farmacológica eficaz e segura para a profilaxia de eventos cardioembólicos em pacientes com FA e trombocitopenia.
