# Supplementary material for: Problemas Relacionados à Trombocitopenia em Pacientes com Fibrilação Atrial Concomitante que Necessitam de Prevenção Antitrombótica: Um Estudo de Coorte Retrospectivo
Source: Arq Bras Cardiol. 2020 Oct 13;115(4):717–8. [Article in Portuguese] doi: 10.36660/abc.20190599 (PMC8386965; doi:10.36660/abc.20190599)
Supplement: Translation

# Translation: Thrombocytopenia-Related Problems in Patients with Concomitant Atrial Fibrillation Requiring Antithrombotic Prevention: A Retrospective Cohort Study

**Authors:** Renato De Vecchis, Andrea Paccone, Silvia Soreca

**Affiliations:** 1 Naples Italy Medical and Polyspecialist Centre, DSB 29 “S. Gennaro dei Poveri Hospital”, Naples - Italy; 2 Bari Italy University of Bari “Aldo Moro” - Department of Cardiology, Bari – Italy

**Keywords:** Cohort Studies, Thrombocytopenia, Atrial Fibrillation, Anticoagulants, Edoxaban, Enoxaparine, Hemorrhage, Stroke, Liver Cirrhosis, Heparin/therapeutic use

## Abstract

Low-dose edoxaban and enoxaparin sodium have been the subject of a retrospective comparison implemented with the propensity score technique in order to mitigate the effects of the differences in the basal clinical features of two cohorts and minimize the risk of bias.

Subsequently, using a Cox proportional-hazards model, the association of each type of therapy with the risk of the composite of all-cause death, stroke/transient ischemic attack, hospitalizations and major bleeding events was assessed. For this analysis, a *p*-value < 0.05 was considered statistically significant. Therapy with enoxaparin and liver cirrhosis as causing thrombocytopenia were associated with increased risk of the composite endpoint (enoxaparin: hazard ratio (HR): 3.31; 95% CI: 1.54 to 7.13; *p* = 0.0023; liver cirrhosis, HR: 1.04; 95% CI: 1.002 to 1.089; *p* = 0.0410). Conversely, edoxaban therapy was significantly associated with decreased risk of the composite endpoint (HR: 0.071; 95% CI: 0.013 to 0.373; *p* = 0.0019). Based on this retrospective analysis, edoxaban at low doses would appear as an effective and safe pharmacological tool for the prophylaxis of cardioembolic events in patients with AF and thrombocytopenia.

## Introduction

A common problem is the presence of atrial fibrillation (AF) in patients suffering from thrombocytopenia, which obviously contraindicates the administration of full-dose anticoagulant drugs.^1^ Moreover, in these cases, the implementation of an AF ablation program involves non-negligible risk, because the first three months following ablation, the so-called “blanking period,” coincide with the need to administer, given the high risk of AF relapses, not only antiarrhythmics, but also anticoagulants at full doses.^2^ Instead, the administration of fractionated low-molecular-weight heparin, namely enoxaparin sodium^3^ at a dose of 4000 I.U. per day, appeared to be a viable option. Alternatively, a widely practiced therapeutic approach was the non-vitamin K antagonist edoxaban^4^ at the prefixed dose of 30 mg per day.

In the present retrospective cohort study, which encompassed a median period of 40 months (interquartile range: from 36 to 48 months), 220 patients were included according to an arbitrary criterion, namely without doing power and sample size calculations. The patients subjected to edoxaban were 90 altogether, whereas 130 patients were assigned to enoxaparin.

The requirements for inclusion in the retrospective study were: moderate thrombocytopenia, defined by a platelet concentration between 99,000 and 30,000 thrombocytes per mm^3^; chronic AF, subject to the rate control strategy; absence of newly diagnosed paroxysmal AF.

Recruitment of cases was based on the constitution of homogeneous groups according to the “propensity score matching” method^5^ to decrease the risk of bias ensuing from the differences in the basal clinical features of the two groups. Patients on unfractionated heparin were paired to patients on a low dose (30 mg per day) of edoxaban with basal clinical features as similar as possible, with a 1:1 ratio. We applied a logistic regression model. Several variables were found to be significantly associated with the probability of belonging to one of the groups, based on a backward stepwise elimination (p = 0.05 cut-off) methodology. The following variables were finally used to calculate the propensity score for each patient: age and care level at index date, previous anticoagulant use, antianginal drug use, insulin use, stroke, hospitalization costs in the baseline period. Patients were matched 1:1 within gender-specific 5-year age groups, based on their propensity score with a maximum allowable difference of 0.001.

The statistical analysis subsequently adopted was the construction of a Cox proportional-hazards regression model. A composite endpoint was chosen including all-cause death, stroke/transient ischemic attack, hospitalizations and major bleeding events. The exposure variables taken into account were low-dose edoxaban therapy, enoxaparin therapy, hypertension, left atrium anteroposterior diameter > 40 mm, left ventricular ejection fraction < 40%, age > 85 years, liver cirrhosis as a cause of thrombocytopenia, Werlhof’s disease as a cause of thrombocytopenia. In all statistical analyses, p value < 0.05 was considered to be statistically significant.

The calculations were made using Excel 2016 (version 16.0, Seattle, WA, USA) as well as MedCalc Version 18.6 (Acacialaan 22, 8400 Ostend, Belgium) and Epi-Info version 7.1.5.0 for Windows (Centers for Disease Control and Prevention, Atlanta, Georgia — USA). Increased risk of being affected by the composite endpoint was associated with exposure to the therapy with enoxaparin (hazard ratio (HR): 3.31; 95% CI: 1.54 to 7.13; p = 0.0023) and liver cirrhosis as causing thrombocytopenia (HR: 1.04; 95% CI: 1.002 to 1.089; p = 0.0410). Even hypertension was associated with increased risk (HR: 1.104; 95% CI: 1.011 to 1.966; p = 0.0477). Conversely, edoxaban therapy was significantly associated with decreased risk of the primary endpoint (HR: 0.071; 95% CI: 0.013 to 0.373; p = 0.0019).

Heparin is a non-negligible cause of thrombocytopenia. This seems to apply also to low-molecular-weight fractioned heparin, i.e., enoxaparin sodium, judging by the results we have found, which advise against the use of enoxaparin therapy in patients with documented thrombocytopenia. Furthermore, the ominous progression of liver cirrhosis might have played a role in causing a significantly higher risk of the composite endpoint. Indeed, the bleeding events due to the rupture of gastroesophageal varices are likely to have played a substantial role in determining the conclusive inference that the composite is unfavorably influenced by liver cirrhosis as an exposure variable. Vice versa, edoxaban therapy at low doses in thrombocytopenic patients has proved to be protective against the composite of all cause-death, stroke/transient ischemic attacks, hospitalizations and major bleeding events. The main limitation of the study is its retrospective nature, which does not allow to draw definitive conclusions about the comparison between enoxaparin and edoxaban due to the possibility of confounding by indication despite the fact that the propensity score matching technique has been adopted as a countermeasure. However, based on our retrospective analysis, edoxaban at low doses would appear as an effective and safe pharmacological tool for the prophylaxis of cardio-embolic events in patients with AF and thrombocytopenia.
